# A novel mutation in the *TG* gene (G2322S) causing congenital hypothyroidism in a Sudanese family: a case report

**DOI:** 10.1186/s12881-018-0588-7

**Published:** 2018-05-02

**Authors:** Y. Watanabe, E. Sharwood, B. Goodwin, M. K. Creech, H. Y. Hassan, M. G. Netea, M. Jaeger, A. Dumitrescu, S. Refetoff, T. Huynh, R. E. Weiss

**Affiliations:** 1 Department of Medicine, University of Miami Miller School of Medicine, 1120 NW 14th St., Room 310F, Miami, FL 33136 USA; 2 Department of Endocrinology and Diabetes, Lady Cilento Children’s Hospital, Brisbane, QLD Australia; 3 Faculty of Medicine, The University of Queensland, Brisbane, QLD Australia; 4 Medical Imaging and Nuclear Medicine, Lady Cilento Children’s Hospital, Brisbane, QLD Australia; 5 Banoon ART & Cytogenetics Centre, Bahrain Defence Force Hospital, West Riffa, Kingdom of Bahrain; 6 Department of Internal Medicine, Radboud University Medical Cente, Nijmegen, The Netherlands; 7 Departments of Medicine, The University of Chicago, Chicago, IL USA; 8 Departments of Medicine, Pediatrics and Genetics, The University of Chicago, Chicago, IL USA; 9 Department of Chemical Pathology, Pathology Queensland, Herston, Brisbane, Queensland Australia

**Keywords:** Congenital hypothyroidism, Goiter, Novel mutations, Thyroglobulin, TG

## Abstract

**Background:**

Congenital hypothyroidism (CH) has an incidence of approximately 1:3000, but only 15% have mutations in the thyroid hormone synthesis pathways. Genetic analysis allows for the precise diagnosis.

**Case presentation:**

A 3-week old girl presented with a large goiter, serum TSH > 100 mIU/L (reference range: 0.7–5.9 mIU/L); free T_4_ < 3.2 pmol/L (reference range: 8.7–16 pmol/L); thyroglobulin (TG) 101 μg/L. Thyroid Tc-99 m scan showed increased radiotracer uptake. One brother had CH and both affected siblings have been clinically and biochemically euthyroid on levothyroxine replacement. Another sibling had normal thyroid function. Both Sudanese parents reported non-consanguinity. Peripheral blood DNA from the proposita was subjected to whole exome sequencing (WES). WES identified a novel homozygous missense mutation of the *TG* gene: c.7021G > A, p.Gly2322Ser, which was subsequently confirmed by Sanger sequencing and present in one allele of both parents. DNA samples from 354 alleles in four Sudanese ethnic groups (Nilotes, Darfurians, Nuba, and Halfawien) failed to demonstrate the presence of the mutant allele. Haplotyping showed a 1.71 centiMorgans stretch of homozygosity in the *TG* locus suggesting that this mutation occurred identical by descent and the possibility of common ancestry of the parents. The mutation is located in the cholinesterase-like (ChEL) domain of TG.

**Conclusions:**

A novel rare missense mutation in the *TG* gene was identified. The ChEL domain is critical for protein folding and patients with CH due to misfolded TG may present without low serum TG despite the *TG* gene mutations.

**Electronic supplementary material:**

The online version of this article (10.1186/s12881-018-0588-7) contains supplementary material, which is available to authorized users.

## Background

Congenital hypothyroidism (CH) has a worldwide incidence of 1:1500 to 1:4000 depending on the TSH cut-off value used in neonatal screening (NS) [1, 2]. Mutations in multiple genes have been shown to cause CH and are grouped into several categories depending on the presence or absence of goiter, level of serum thyroglobulin (TG), and other clinical characteristics [3]. TG is synthesized by the thyroid gland and its primary functions include iodide storage and thyroid hormonogenesis. Thyroxine (T_4_), the predominant form of thyroid hormone (TH), is produced by iodination of select tyrosine residues and coupling pairs of doubly iodinated tyrosines within TG molecules. TG is essential for TH synthesis and homozygous or compound heterozygous mutations of the *TG* gene can result in permanent CH. To date, 117 *TG* gene mutations have been reported [4–6]. Most cases with CH due to *TG* gene mutations show decreased serum TG levels.

We report an unusual case identified in Australia involving a Sudanese family with familial CH presenting with goiter and without low serum TG level.

## Case presentation

### Clinical report

**Fig. 1:**
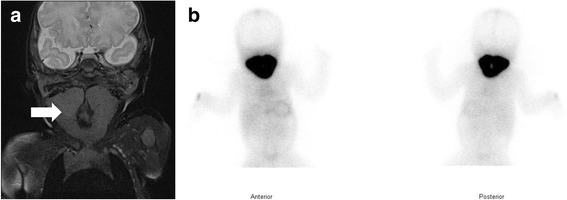
Radiological investigations of the proposita at three weeks of age. **a** Magnetic Resonance Imaging of the neck showing significant enlargement and extension of the thyroid gland. The thyroid encircles the laryngeal and tracheal lucency, extending posteriorly almost to the midline. Inferiorly, it extends posterior to the clavicle with no retrosternal extension. **b** Tc-99 m pertechnetate thyroid scan demonstrating significantly increased radiotracer uptake

The CARE guidelines were followed in this case. A 3-week old girl born to Sudanese parents in Brisbane, Australia, presented with a large goiter causing upper airway obstruction and producing stridor. Her thyroid stimulating hormone (TSH) on NS was 14 mIU/L (cut-off range for notification: > 13 mIU/L). The result was communicated to the relevant clinicians but, unfortunately, appropriate follow-up had not occurred. At 3 weeks of age when she presented with goiter, serum analysis showed a TSH > 100 mIU/L (reference range: 0.7–5.9 mIU/L) with low free T_4_ of < 3.2 pmol/L (reference range: 8.7–16 pmol/L) and high TG of 101 μg/L (reference range for all age: 1.6–59.9 μg/L). The urinary iodine concentration was 0.63 μmol/L, suggesting that she had mild iodine deficiency. Thyroid ultrasound and MRI (Fig. 1a) showed both lobes of the thyroid gland to be significantly enlarged. Nuclear scan following injection of 42 MBq Tc-99 m pertechnetate showed a large goiter with homogeneous markedly increased radiotracer uptake (Fig. 1b). Levothyroxine replacement was started based on the diagnosis of CH as standard of care.

**Fig. 2:**
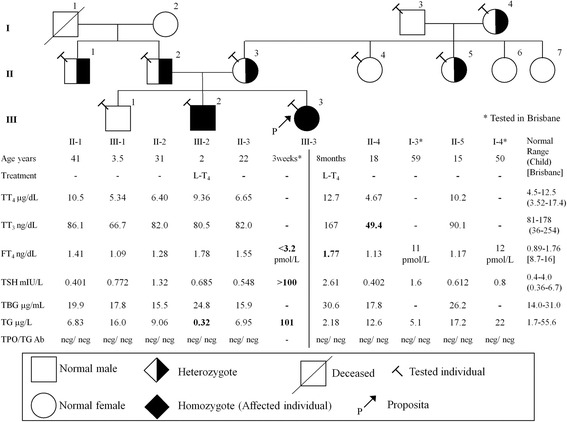
Pedigree of the family and results of thyroid function tests. Each generation corresponds to a roman number. Arabic numbers above each symbol identify the subjects. Laboratory data are aligned below each symbol. Abnormal values are in bold type. Abbreviations: L-T_4_; levothyroxine, TT_4_; total thyroxine, TT_3_; total triiodothyronine, FT_4_; free thyroxine, TSH; thyroid-stimulating hormone, TBG; thyroxine binding globulin, TG; thyroglobulin, TPO Ab; anti-TPO antibody, TG Ab; anti-thyroglobulin antibody. The International System of Units: TT_4_; μg/dL = 12.87 nmol/L, TT_3_; ng/dL = 0.0154 nmol/L, FT_4_; ng/dL = 12.87 pmol/L, TBG; μg/mL = 0.0185 μmol/L

**Fig. 3:**
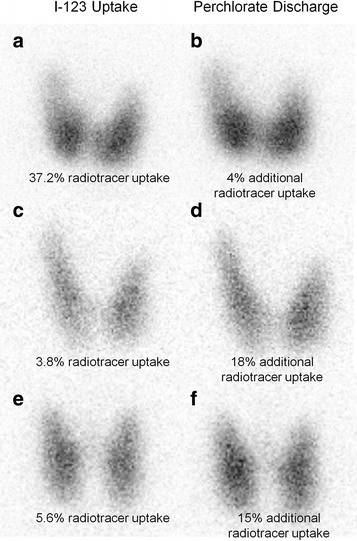
I-123 and perchlorate discharge thyroid scans for the proposita’s brother (**a** & **b**), father (**c** & **d**), and mother (**e** & **f**). All subjects demonstrated no discharge following perchlorate administration consistent with preservation of the organification process

Her brother was also found to be hypothyroid based on NS, with an elevated TSH of 60 mIU/L. He had been on levothyroxine replacement since that time, with no concerns regarding growth or development. His thyroid ultrasound and nuclear scan at 2 years of age showed a eutopic thyroid gland with increased nuclear tracer uptake of 5.2% (normal range 1–4%) following withdrawal of levothyroxine replacement for 5 days. Both siblings had TSH and TH concentrations in the reference ranges while under levothyroxine replacement and with dose adjustment and they have been clinically euthyroid (Fig. 2). The proposita’s TG decreased to 2.2 μg/L and the affected brother had low TG of 0.3 μg/L. Other members of the family had no symptoms and their thyroid tests were normal (Fig. 2). Subsequent perchlorate discharge studies were performed on the affected brother following withdrawal of levothyroxine replacement for 3 weeks [TSH 180 mIU/L (reference range: 0.7–4.0 mIU/L), FT_4_ 4.4 pmol/L (reference range: 7.5–17 pmol/L), TG 9.6 μg/L] and on both parents. The results demonstrated increased uptake of radiotracer in the brother and normal tracer uptake in the parents with no washout of I-123 following perchlorate administration (Fig. 3). This suggests preservation of the organification process [7]. Both maternal and paternal families are non-consanguineous and of Sudanese origin (maternal grandfather; Gogrial and maternal grandmother and paternal grandparents; Aweil East, of South Sudan, respectively). There was no history of thyroid disorders in either family.

### Molecular genetics

**Fig. 4:**
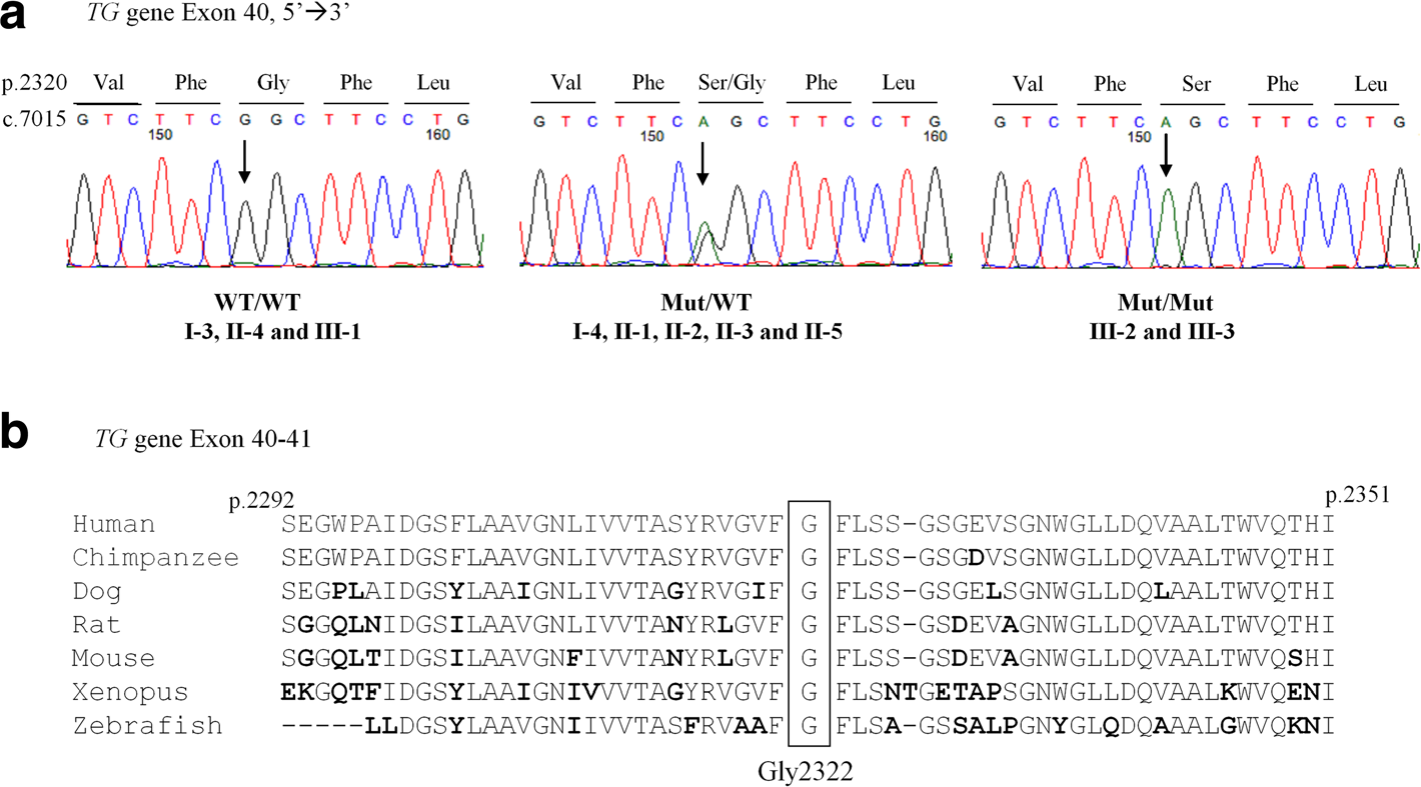
**a** Direct sequencing for normal (Wild type/Wild type), heterozygote (Mutant/Wild type) and homozygote (Mutant/Mutant) covering the region of the mutation in exon 40 of the *TG* gene (c.7021G > A, pGly2322Ser). **b** Alignment of the *TG* amino acid sequence encoded by exons 40 and 41 containing the mutant Gly2322 in various species including mammals, amphibian and fish. Amino acid differences are in bold type. Abbreviations: WT; Wild type, Mut; Mutant

**Fig. 5:**
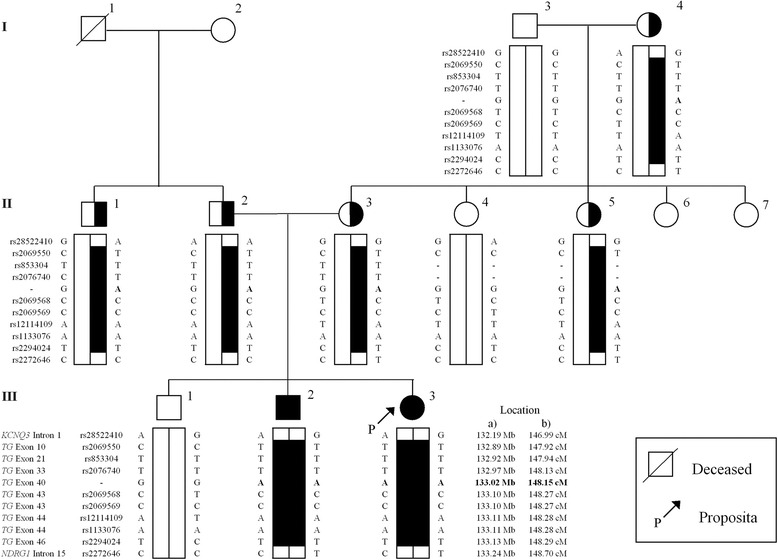
Haplotypes present in the family members using single nucleotide polymorphic markers. Black filled, semi-round or semi-square and open symbols indicate affected individuals, unaffected carriers and wild type, respectively. The inherited portion of the disease associated haplotype is indicated in blackened bar. a) Marker location on the human genome reference sequence (NCBI Build 38). b) Marker location on the international HapMap project

Genomic DNA was extracted from peripheral blood leukocytes as previously described [8]. The proposita’s DNA was submitted to whole exome sequencing. A panel of 50 genes related to thyroid development, function, serum and cell TH transport and hormone synthesis was evaluated (Additional file 1: Table S1). This led to the identification of a novel homozygous missense mutation in exon 40 of the *TG* gene: c.7021G > A, p.Gly2322Ser (numbering excludes 19 amino acid signal peptide). All exonic variants in the *TG* gene identified in the proposita are summarized in Additional file 2: Table S2. Sanger sequencing confirmed that the affected brother (III-2) was homozygous for the same mutation while the parents (II-2 and II-3) and maternal grandmother (I-4) were heterozygous for this mutation, indicating that they were unaffected carriers (Fig. 4a). The other brother (III-1) and the maternal grandfather (I-3) had the wild-type genotype and normal thyroid function tests (Fig. 2 and 4a). This variant was not present in the Genome Aggregation Database (gnomAD) used for analysis. Since the database did not likely include a large number of Sudanese individuals, 354 alleles from 4 Sudanese ethnic groups (Nilotes, Darfurians, Nuba, and Halfawien) [9] were also used for analysis. The *TG* mutation identified in our family was not present in the 177 Sudanese individuals, suggesting that the variant is rare in the Sudanese as well. Single nucleotide polymorphisms (SNPs) markers with high allele frequencies in the *TG* locus and markers in the flanking genes were genotyped to reconstruct the haplotype for the region (8q24.22) (Fig. 5). The haplotype associated with CH present in both parents was identical for a stretch of ~ 1.71 centiMorgans (cM). The G2322S *TG* mutation affects a highly conserved amino acid in various species (Fig. 4b) and functional in silico prediction algorithms suggest that this mutation is deleterious (SIFT; 0.001, deleterious and PolyPhen2; 1.0, probably damaging).

## Discussion and Conclusions

A novel homozygous *TG* gene mutation was identified in two children with CH and goiter. The unrelated parents are heterozygous for this “rare” mutation and, surprisingly, report non-consanguinity. Haplotype analysis of the SNP makers revealed that the haplotype containing the mutated allele was homozygous over the entire *TG* locus, spanning 1.71 cM (Fig. 5). This is consistent with the autosomal recessive pattern of inheritance [10]. These results suggest that the mutation occurred identical by descent [11] and that the parents have distant common ancestry [12]. This is likely because Sudan is known for high prevalence of consanguineous marriage [13] and it has been reported that marriage between 1st cousins accounted for more than 40% marriages [14]. DNA obtained from a Sudanese cohort [9] was analyzed by Sanger sequencing to confirm the frequency of the mutation in the population. However, none of 177 individuals had the mutated allele, indicating the minor allele frequency (MAF) for this variant to be less than 0.3% in the Sudanese population.

**Fig. 6:**
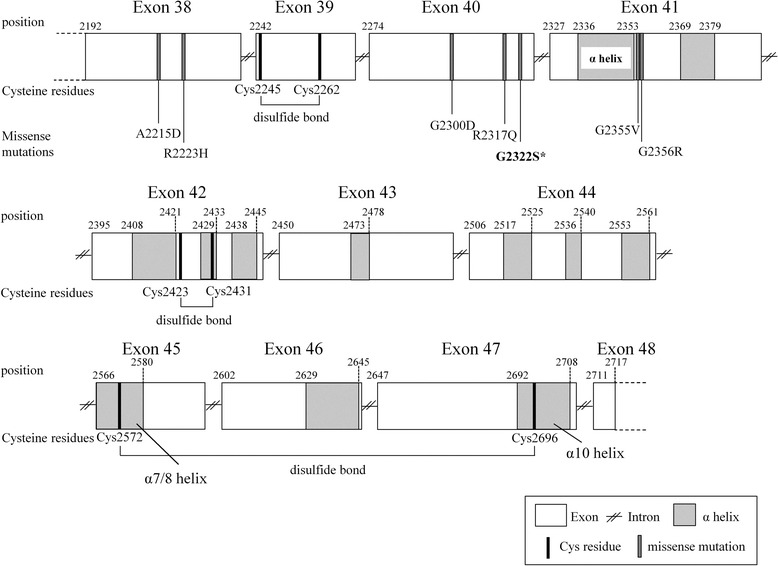
Schematic representation of ChEL domain in the *TG* gene. Amino acid numbering is indicated above TG structure. α helix motifs are indicated in gray boxes with diagonal lines. Cystein residues are indicated in black lines. Pathogenic missense mutations are indicated in gray lines. *Mutation in the present case

The TG protein is composed of three consecutive cysteine (Cys) repeat domains (domains I, II, and III) forming many disulfide bonds. These Cys repeat-domains are followed by the cholinesterase-like (ChEL) domain [4]. Although the TG protein contains 120 Cys, the ChEL domain contains only six Cys. While the mutation in the present case does not directly substitute a Cys residue, it is located in the ChEL domain and is similar to six pathogenic missense mutations previously reported in the domain. Two mutations in the domain (p.A2215D and p.R2223H) occur before the first disulfide bond of ChEL, whereas the remaining four mutations (p.G2300D, p.R2317Q, p.G2355 V, and p.G2356R) fall between the first and second disulfide bonds. The G2322S variant in the present case also resides between the first and second disulfide bonds of the ChEL domain (Fig. 6). We used JPred 4 server for prediction of the secondary structure of the TG [15]. Although the first loop created by the first and second disulfide bonds in the ChEL domain are located away from the α7/8 and α10 helices, associated with TG dimerization, or any other α helix motif, the first loop is considered to be important for ChEL folding [4]. Interestingly, all pathogenic missense mutations in the ChEL domain are not located in the α helix, however they have been shown to result in CH. Animal studies confirmed that a misfolded TG resulted from similar variants in the ChEL domain. Introduction of p.L2263P in a mouse produced congenital goiter demonstrating that the mutation permitted full-length synthesis of TG but impaired folding necessary for TG homodimerization and transport from the endoplasmic reticulum (ER) [16, 17]. Another rodent, the *rdw* rat, harboring the *TG* p.G2300R mutation exhibited abnormal folding of TG resulting in an extended α helix within the ChEL domain and retention of TG within the ER lumen [18]. The p.G2322S mutation in the present case is located near these mutations, suggesting that TG folding, transport and secretion might be similarly impaired.

Most cases with CH due to the *TG* gene mutations show decreased serum TG levels before TH replacement and it is a key factor for the diagnosis of TG defects [6, 19]. Interestingly, the present case had a TG of 101 μg/L at diagnosis. Reference range of serum TG for infants have not been established. Depending on the platform of analysis, the upper limit of normal for infants between 15 and 180 days was from 43.37 to 147.28 μg/L [20]. Due to lack of a specific TG reference range for infants using our method (IMMULITE 2000 ®; Siemens, Germany), the TG value of the present case may be high normal, but definitely above values reported in individuals with the *TG* gene mutations. Moreover, her TG decreased as TSH fell. The affected brother (III-2) had the same mutation in the *TG* gene, although his TG before levothyroxine replacement therapy was unknown. Among 61 cases in which serum TG values without TH replacement were reported, only three cases, beside the present case, showed increased TG (Table 1) [21–23]. The *TG* mutations p.C1977S and p.A2215D have been reported in multiple cases, and levels of TG in the affected, not included in the list, were normal or low. As with the p.G2322S or p.A2215D in the ChEL domain, the p.C1977S located in the Cys repeat domain III was also associated with misfolding [21, 24]. In general, unfolded protein is blocked from being transported within a cell and then refolded or degraded by chaperone proteins through an intracellular signaling pathway called unfolded protein response (UPR) [24]. Interestingly, absence of the chaperone protein activation was observed in the p.C1977S case with hyperthyroglobulinemia [24]. Thus, incomplete UPR may be associated with secretion of some mutated TG without retention in the ER resulting in higher serum TG. The initial serum TG level of the present case was 101 μg/L. In contrast, CH due to TPO deficiency, in 63 published cases in whom the *TG* gene was normal had on the average serum TG of more than 1000 μg/L before TH replacement [25–27]. Therefore the *TG* mutation in the present case resulted in a lesser serum TG level than that observed in CH due to *TPO* mutations but higher than that of patients with *TG* gene mutations in the majority of whom serum TG is undetectable or less than 5 μg/L [19]. The higher level of serum TG in the present case suggests reduced secretion or rapid degradation of a misfolded molecule. As mentioned above, several other cases of *TG* gene mutations manifesting serum TG levels above 5 μg/L have been reported [21–23].

**Table 1:** Clinical characteristics of the CH cases due to the *TG* gene mutations with detectable TG levels

Position				Age Gender	Goiter	TSH (mIU/L) [normal]	TT_4_ (μg/dL) [normal]	FT_4_ (pmol/L) [normal]	TG (μg/L) [normal]	Antibodies	References
Exon	Nucleotide	Amino Acid	TG domain								
33	c.5985 T > A	p.C1977S	Repeat domain III	adult F	+	1.7 [0.1–4.0]	N/A	9.8 [8–28]	181 [15–50]	negative	[21]
33	c.5985 T > A	p.C1977S	Repeat domain III	adult F	+	1.2 [0.1–4.0]	N/A	9.1 [8–28]	117 [15–50]	negative	[21]
38	c.6701C > A	p.A2215D	ChEL	34 yrs. M	+	4 [0.5–4.5]	6 [4–12]	N/A	29.2 [1.5–15]	N/A	[22, 23]
40	c.7021G > A	p.G2322S	ChEL	3 wks F	+	> 100 [0.7–5.9]	< 3.2 [8.7–16]	N/A	101 [N/A]	negative	III-3, this report

All patients were without TH replacement at the thyroid function tests

Abbreviations: *TG* thyroglobulin, *TSH* thyroid-stimulating hormone, *TT*_*4*_ total thyroxine, *FT*_*4*_ free thyroxine, *N/A* not available, *L-T*_*4*_; levothyroxine, *ChEL* cholinesterase-like, *F* Female, *M* Male, *yrs* years, *wks* weeks

The International System of Units: TT_4_; μg/dL = 12.87 nmol/L

The affected sibling of the proposita homozygous for the *TG* mutation had an elevated TSH on NS whereas the proposita (also homozygous for the same mutation) did not. “Delayed TSH rise” has been reported in pre-term and low-birth weight newborns and also some cases with thyroid dyshormonogenesis [28–30]. Incomplete development of hypothalamic-pituitary axis or acute iodine overload is possible cause of the phenomenon. However, the precise pathophysiological mechanisms are still unclear. The delayed TSH rise in the proposita may be due to an unknown genetic cause or an environmental factor such as iodine exposure.

In conclusion, a novel homozygous mutation in the *TG* gene was identified. The mutation was not found in four Sudanese groups of different ethnic origin (*n* = 177). The mutation is located in the ChEL domain of the TG which is essential for protein folding. Patients with CH due to misfolded TG may present without low serum TG.

## Additional files

Additional file 1: **Table S1**. Genes related to thyroid disorder. 50 genes related to thyroid development, function, serum and cell TH transport and hormone synthesis. (XLSX 30 kb)

Additional file 2: **Table S2.** Exonic variants in the *TG* gene identified in the proposita by whole exome sequencing. All exonic variants in the *TG* gene identified in the proposita. (XLSX 41 kb)

## Abbreviations

CH: Congenital hypothyroidism
ChEL: Cholinesterase-like
cM: CentiMorgans
Cys: Cysteine
ER: Endoplasmic reticulum
gnomAD: Genome Aggregation Database
L-T_4_: Levothyroxine
MAF: Minor allele frequency
NS: Neonatal screening
SNP: Single nucleotide polymorphism
T_3_: Triiodothyronine
T_4_: Thyroxine
TG: Thyroglobulin
TH: Thyroid hormone
TSH: Thyroid stimulating hormone
UPR: Unfolded protein response
